# Serum S100B, Lactate Dehydrogenase and Brain Metastasis Are Prognostic Factors in Patients with Distant Melanoma Metastasis and Systemic Therapy

**DOI:** 10.1371/journal.pone.0081624

**Published:** 2013-11-28

**Authors:** Benjamin Weide, Sabina Richter, Petra Büttner, Ulrike Leiter, Andrea Forschner, Jürgen Bauer, Laura Held, Thomas Kurt Eigentler, Friedegund Meier, Claus Garbe

**Affiliations:** 1 Department of Dermatology, University Medical Center, Tübingen, Germany; 2 German Cancer Research Center (DKFZ), Heidelberg, Germany; 3 German Cancer Consortium (DKTK), Heidelberg, Germany; 4 Skin Cancer Research Group, School of Public Health, Tropical Medicine and Rehabilitation Sciences, James Cook University, Townsville, Australia; The University of Queensland, Australia

## Abstract

**Background:**

Prognostic factors of melanoma with distant metastasis and systemic treatment are only poorly established. This study aimed to analyse the impact of S100B, lactate dehydrogenase (LDH) and the type of treatment on survival in advanced patients receiving systemic treatment.

**Patients and Methods:**

We analysed overall survival of 499 patients from the university department of dermatology in Tuebingen, Germany, with unresectable melanoma at the time point of initiation of first-line systemic therapy. Only patients who started treatment between the years 2000 and 2010 were included. Disease-specific survival was calculated by bivariate Kaplan Meier survival probabilities and multivariate Cox hazard regression analysis.

**Results:**

In univariate analysis LDH, S100B, the site of distant metastasis (soft tissue vs. lung vs. other visceral), the presence of brain metastases and the type of treatment (monochemotherapy, polychemotherapy, immunotherapy or biochemotherapy) were associated with overall survival (all p<0.001). In multivariate analysis LDH (Hazard ratio [HR] 1.6 [1.3–2.1]; p<0.001), S100B (HR 1.6 [1.2–2.1]; p<0.001) and the presence of brain metastases (HR 1.5 [1.1–1.9]; p = 0.009), but not the type of treatment had significant independent impact. Among those factors normal S100B was the best indicator of long-term survival, which was 12.3% after 5 years for this subgroup.

**Conclusion:**

Serum S100B is a prognostic marker predicting survival at the time of initiation of first-line treatment in unresectable melanoma patients. Compared to the other independent factors LDH and the presence of brain metastases it is most appropriate to predict long-term survival and requires further prospective investigation in patients treated with new and more potent drugs in metastatic melanoma.

## Introduction

The prognosis of advanced melanoma patients is poor. Long term survival can be observed after complete metastasectomy of distant metastases with 5-years survival rates up to 41% [1], [2] but is rarely observed in patients with unresectable disease after systemic therapies [3]. For decades it was questionable whether the natural course of disease can be improved by available systemic treatments at all. Overlapping survival curves were observed in a number of randomized clinical trials (RCTs) testing different drugs and clinical responses were only eventually observed. In a meta-analysis of 41 trials conducted before 2003 the outcome after systemic therapies was investigated comparing mono-, polychemotherapy and immunotherapy, and biochemotherapy. While the response rate was higher in patients receiving poly- or bio-chemotherapy regimen, overall survival was not affected according to the treatment category [4]. Before 2010, an improved overall survival could not be demonstrated by any systemic drug treatment in randomized controlled clinical trials (RCTs) and prognosis of patients with unresectable distant metastases was mainly defined by the serum lactate dehydrogenase (LDH) and the localization of distant metastases. Both factors were intensively studied before start of systemic therapy [5]–[7]. Based on these studies LDH was the first serum biomarker to be included in the American Joint Committee on Cancer (AJCC) staging system for patients with distant metastases since 2001 in addition to the pattern of involved organs [8]. Both factors remain the most important strata in RTCs and are used to classify AJCC stage IV into the M categories M1a (soft tissue metastasis), M1b (pulmonary involvement), and M1c (involvement of other visceral organs or elevated LDH) [9].

In addition to LDH, S100B is an independent prognostic serum marker at the time of stage IV diagnosis as reported by us before and can be useful to select patients for complete metastasectomy.[3] However, its value at later time-points, analysing patients with unresectable disease and increasing tumour-load, remains inconclusive. Before start of systemic treatment multivariate analyses comparing S100B and LDH and considering established clinical data have only been reported in small cohorts with conflicting results [10]–[14].

In the present study we investigated prognostic factors in a retrospective cohort of 499 institutional melanoma patients who received first-line systemic treatment between 2000 and 2010. The main aims were (a) to analyse if the type of systemic treatment is relevant for survival of unselected patients and (b) to clarify the prognostic impact of the serum marker S100B at the initiation of first line systemic therapy compared to LDH.

## Methods

### Ethics statement

All patients had given their written informed consent to have clinical data recorded by the Central Malignant Melanoma Registry (CMMR) registry. The institutional ethics committee Tübingen approved the study (ethic vote 449/2013R).

### Patients

Patients from the university department of dermatology in Tuebingen, Germany, with cutaneous or unknown primary melanoma and distant metastasis were identified in the Central Malignant Melanoma Registry (CMMR) database which prospectively records patients from more than 60 dermatological centres in Germany. Next, those with the first systemic treatment for non-resectable melanoma initiated between 2000 and 2010, and available follow-up data were selected, resulting in a final sample size of 499 after individual file review. All patients had given their written informed consent to have their data recorded by the CMMR. The aims and methods of data collection by the CMMR have previously been reported in detail [15].

Data obtained for each patient included gender, the date of the last follow-up, and the date and cause of death, if applicable. The following characteristics of the primary tumour were analyzed: anatomical localization (axial vs. extremities, or unknown primary), Breslow's tumour thickness, Clark's level of invasion, ulceration, histopathological subtype (superficial spreading melanoma [SSM] vs. nodular melanoma [NM] vs. lentigo maligna melanoma [LMM] vs. acral lentiginous melanoma [ALM]). At the time of start of systemic treatment the following variables were evaluated: age, site of visceral involvement (soft tissue metastasis vs. pulmonary involvement vs. other visceral sites), serum LDH (normal vs. >upper limit of normal [ULN]) and S100B (normal vs. >ULN). S100B was detected using the Sangtec S100 ELISA (Diasorin Inc., Stillwater, USA; ULN = 0.15 µg/l) until December 2003 and thereafter by the Elecsys S100 electrochemiluminescence immunoassay (Roche Diagnostics AG, Rotkreuz, Switzerland, ULN = 0.10 µg/l) according to the instructions of the manufacturers. The treatment was categorized as either monochemotherapy, polychemotherapy (any combination regimen including at least 2 cytotoxic drugs), immunotherapy, biochemotherapy (combination of chemotherapy and immunotherapy), or others (not assignable to any of the listed categories).

### Statistical analysis

The categorization of the body site und tumor thickness of the primary melanoma was performed as described before by studies based on the AJCC database [16]. Categorized variables were dummy coded to adhere to the linearity assumption of multivariable regression analysis. Follow-up time was defined from the start of systemic treatment to the date of last follow-up or death. Estimates of cumulative survival probabilities according to Kaplan-Meier were described together with 95% confidence intervals (CIs) and compared using two-sided log-rank test statistics. Median survival times (MST) are presented. For the analysis of overall survival patients who were alive at the last follow-up were censored, while patients who had died were considered an “event”.

Multivariable Cox proportional hazard models were used to determine independent prognostic factors. Patients with missing values were excluded from multivariable testing. Proportional hazard assumptions were checked graphically plotting the log(-log(survival)) versus the log of survival time. Models were initially built as hierarchical structures including interactions; although no interaction term was found statistically significant. Models were compared using the Chi-square distributed differences of the log likelihood of each model. Forward and backward stepwise procedures of the multivariable modelling process were conducted. Results of the Cox models were described by means of hazard ratios together with 95% CIs, p-values were based on the Wald test.

Confounding was assessed by checking the effect of each remaining non-significant variable, which was not in a model, on factors in the model. If changes in the estimate of factors in the model of 5% or more occurred the variable was considered a confounder. Throughout the analysis, p values less than 0.05 were considered as statistically significant. All statistical analyses were carried out using the SPSS Version 20 (IBM SPSS, Chicago, Illinois, USA).

## Results

### Description of sample

Patients' characteristics are shown in Table 1. A total of 499 patients (58.5% male) were included in the survival analysis at the start of the first systemic treatment for non-resectable stage IV melanoma. The median age was 60 years (inter quartile range [IQR] 49–70 years). The median follow-up for patients who died was 10 months and 27 months for patients who were alive at the last date of observation. The median survival time according to Kaplan Meier (MST) was 9 months. Cumulative survival rates were 40.0% (1-year), 15.9% (2-years), and 6.2% (5-years). Based on the site of distant metastases the assignment to the M category was M1c in 65.9%, M1b in 23.6% and M1a in 10.4% of patients. 109 patients (21.8%) had brain metastases. An elevated S100B or LDH serum level was observed in 69.2% and 40.0%, respectively. The first systemic therapy was a monochemotherapy in the majority of patients (58.9%), a biochemotherapy, polychemotherapy or immunotherapy was applied in 18.0%, 16.6%, and 6.5% of patients.

**Table 1:** Descriptive statistics and survival rates according to Kaplan-Meier.

Prognostic factor		n	%	% dead	1-year survival rate [95-CI#] (%)			2-years survival rate [95-CI#] (%)			p*
Sex	Male	292	58.5	91.1	38.0	32.3	43.6	13.7	9.6	17.8	0.188
	Female	207	41.5	89.9	42.8	35.9	49.6	19.0	13.5	24.5	
Age at time of systemic therapy	<50 years	128	25.7	92.2	40.4	31.7	49.0	14.8	8.5	21.1	0.914
	50–59 years	117	23.4	89.7	38.5	29.5	47.5	14.5	7.8	21.2	
	60–69 years	128	25.7	91.4	39.4	31.0	47.8	16.4	9.9	22.9	
	≥70 years	126	25.3	88.9	41.6	33.0	50.2	17.8	10.9	24.7	
Primary tumor	Occult primary	98	19.6	91.8	44.3	34.5	54.1	13.9	6.8	21.0	0.790
	Apparent	401	80.4	90.3	39.2	34.3	44.1	16.4	12.7	20.1	
Body site of primary	Axial	234	58.4	91.0	37.5	31.2	43.8	16.0	11.3	20.7	0.435
	Extremities	167	41.6	89.2	41.5	34.1	49.0	16.9	11.2	22.6	
	Missing	98		91.8	43.4	33.6	53.2	13.9	6.8	21.0	
Histologic subtype of primary	SSM	176	51.5	90.3	37.3	30.1	44.6	17.0	11.3	22.7	0.597
	Nodular	107	31.3	89.7	42.3	32.9	51.7	18.6	11.0	26.2	
	LMM	16	4.7	93.8	31.3	8.6	54.0	12.5	0	28.8	
	ALM	43	12.6	93.0	50.4	35.3	65.5	14.4	3.8	25.0	
	Missing data	157		90.4	39.4	31.8	47.0	13.5	8.0	19.0	
Breslow's thickness of primary	≤1 mm	61	15.9	93.4	30.1	18.5	41.7	7.1	0.4	13.8	0.157
	1.01–2 mm	108	28.2	88.9	31.6	22.8	40.4	16.1	9.0	23.2	
	2.01–4 mm	116	30.3	91.4	46.4	37.4	55.4	19.2	12.0	26.5	
	>4 mm	98	25.6	89.8	44.5	34.7	54.3	19.0	11.2	26.8	
	Missing data	116		90.5	42.6	33.6	51.6	14.4	7.7	21.1	
Ulzeration of primary	Yes	160	47.5	89.4	41.3	33.7	48.9	16.7	10.8	22.6	0.706
	No	177	52.5	89.8	40.2	33.0	47.5	19.4	13.5	25.3	
	Missing data	162		92.6	38.5	31.1	46.0	11.0	6.1	15.9	
Clark's level of invasion	Level I–III	65	20.3	90.8	30.8	19.6	42.0	13.7	5.3	22.1	0.422
	Level IV	212	66.3	91.5	39.6	32.9	46.3	17.0	11.9	22.1	
	Level V	43	13.4	83.7	43.3	28.4	58.2	19.2	7.2	31.2	
	Missing data	179		91.1	43.1	35.9	50.4	14.7	9.4	20.0	
Site of distant metastasis	Soft tissue only	52	10.4	84.6	54.8	41.1	68.5	24.2	12.2	36.2	<0.001
	Lung	118	23.6	93.2	53.4	44.4	62.4	20.9	13.5	28.4	
	Other visceral	329	65.9	90.6	32.8	27.7	37.9	12.8	9.1	16.5	
Presence of brain metastases	No	390	78.2	89.0	44.9	40.0	49.8	18.5	14.6	22.4	<0.001
	Yes	109	21.8	96.3	22.5	14.7	30.3	6.6	1.9	11.3	
LDH	Elevated	175	40.0	93.1	20.1	14.0	26.2	6.9	3.0	10.8	<0.001
	Normal	263	60.0	87.5	50.6	44.5	56.7	21.3	16.2	26.4	
	Missing	61		96.7	50.8	38.3	63.3	18.0	8.4	27.6	
S100B	Elevated	279	69.2	93.5	30.3	24.8	35.8	8.9	5.4	12.4	<0.001
	Normal	124	30.8	84.7	52.7	43.9	61.5	25.3	17.5	33.1	
	Missing	96		90.8	51.6	41.6	61.6	23.7	15.1	32.3	
First systemic therapy	Monochemotherapy	291	58.9	90.4	41.4	35.7	47.1	15.9	11.6	20.2	<0.001
	Polychemotherapy	82	16.6	98.8	19.5	10.9	28.1	3.7	0	7.8	
	Immuntherapy	32	6.5	84.4	46.9	29.7	64.2	25.0	9.9	40.1	
	Biochemotherapy	89	18.0	86.5	51.2	40.8	61.6	24.6	15.6	33.6	
	Other or missing data	5									

# 95%-CI: 95% confidence interval;

*two-sided log-rank excluding missings.

### Survival analysis according to Kaplan-Meier

**Figure 1:**
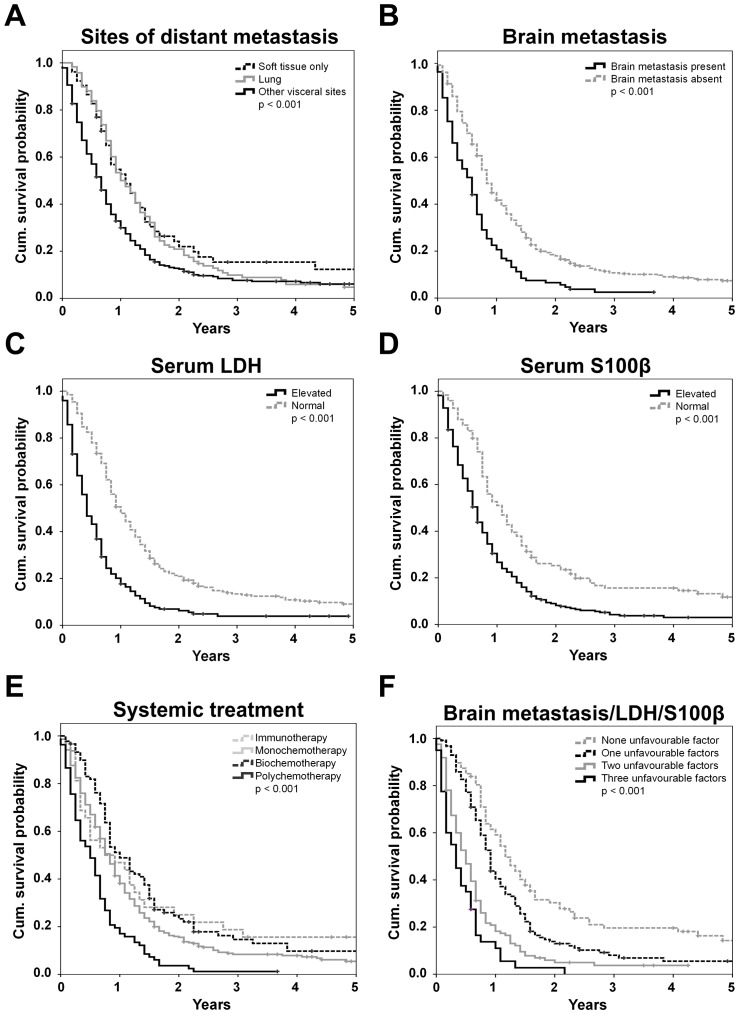
Univariate analysis of 499 patients. Kaplan Meier survival curves according to (A) the site of distant metastasis, (B) the presence of brain metastases, (C) the LDH serum level, (D) the S100B serum level, (E) the type of treatment, and (F) the number of applicable unfavourable independent prognostic factors according to the multivariate analysis (S100B, LDH, presence of brain metastasis). Censored events are indicated by vertical lines.

Both factors considered in the AJCC-staging system for patients with distant metastasis (site of distant metastases and LDH) were strongly correlated with survival before receiving the first systemic therapy. If distant lesions were limited to soft tissue or the lung MST was 13 or 12 months, respectively, while it was shorter for patients with other visceral lesions (8 months; *p*<0.001; Figure 1A). The probability to be alive one year after start of systemic treatment was twice as high for patients without cerebral involvement compared to those with brain metastasis (44.9 vs. 22.5; log rank p<0.001; Figure 1B). Both analysed serum markers were significantly (each log rank p<0.001) associated with survival (Figure 1C, 1D). In patients with an elevated LDH levels MST was 5 months, representing the most powerful indicator for poor MST among all analysed factors. Differences in prognosis were likewise observed according to the type of treatment, with poorest MST of 6 months in patients receiving polychemotherapy and favourable MST of 12 months for those treated with biochemotherapy. Immunotherapy was associated with a MST of 9 months but the chance for long term benefit was highest for those patients with a probability of 15.6% to survive 5 years or longer (Figure 1E). No differences in prognosis were evident for age, gender, or histopathological characteristics of the primary melanoma. An overview over one- and two-year survival rates is presented in Table 1.

### Multivariable Cox Proportional Hazard Analysis

Three different approaches of multivariate testing were performed to compare the relative impact of prognostic factors (Table 2).

**Table 2:** Multivariate analysis for disease-specific death.

Prognostic factor	Sample size	% Dead	Relative risk (95% CI)#	p-value
**Model 1 (n = 438)** *				
**M category**				
M1a	44 (10.0%)	84.1%	1	
M1b	97 (22.1%)	91.8%	1.2 (0.83, 1.8)	P = 0.324
M1c	297 (67.8%)	89.9%	1.5 (1.1, 2.1)	P = 0.021
**Lactate Dehydrogenase**				
Normal	263 (60.0%)	87.5%	1	
Elevated	175 (40.0%)	93.1%	2.1 (1.7, 2.6)	P<0.001
**Model 2 (n = 377)** **				
**Lactate Dehydrogenase**				
Normal	228 (60.5%)	88.2%	1	
Elevated	149 (39.5%)	93.3%	1.7 (1.4, 2.2)	P<0.001
**S100B**				
Normal	117 (31.0%)	83.8%	1	
Elevated	260 (69.0%)	93.1%	1.6 (1.2, 2.1)	P<0.001
**Cerebral metastases**				
No	293 (77.7%)	88.4%	1	
Yes	84 (22.3%)	96.4%	1.6 (1.2, 2.1)	P = 0.001
**Model 3 (n = 372)** ***				
**Lactate Dehydrogenase**				
Normal	225 (60.5%)	88.0%	1	
Elevated	147 (39.5%)	93.9%	1.6 (1.3, 2.1)	P<0.001
**S100B**				
Normal	116 (31.2%)	83.6%	1	
Elevated	256 (68.8%)	93.4%	1.6 (1.2, 2.1)	P<0.001
**Cerebral metastases**				
No	288 (77.4%)	88.5%	1	
Yes	84 (22.6%)	96.4%	1.5 (1.1, 1.9)	P = 0.009
**First systemic therapy**				
Monochemotherapy	229 (61.6%)	90.0%	1	
Polychemotherapy	65 (17.5%)	100%	1.3 (0.97, 1.8)	P = 0.074
Immunotherapy	24 (6.5%)	87.5%	0.81 (0.51, 1.3)	P = 0.363
Biochemotherapy	54 (14.5%)	81.5%	0.75 (0.54, 1.04)	P = 0.085

# 95% CI = 95% confidence interval;

*61 patients had unknown values for LDH and were excluded; no confounding and no significant interaction was detected.

**122 patients had unknown values for LDH and/or S100 and were excluded; the model was adjusted for the confounding effects of M Stage IV; no significant interaction was detected.

***127 patients had unknown values for LDH and/or S100 and/or could not be aligned to one of the 4 treatment categories and were excluded; the model was adjusted for the confounding effects of the M category; no significant interaction was detected.

First, we aimed to focus on the prognostic factors considered in the AJCC staging classification. The treatment category, S100B and presence of brain metastases were therefore not considered in the first model. As expected, the site of distant metastases and LDH, had both independent impact on prognosis. An elevated LDH and the presence of visceral metastases other than lung and/or soft-tissue increased the risk to die from disease by 2.1 [1.7–2.6];p<0.001 or 1.5 [1.1–2.1];p = 0.021, respectively.

In the second model S100B and the presence of brain metastases were likewise considered. We observed an independent impact of S100B on prognosis (HR1.6 [1.2–2.1]; p<0.001) in addition to LDH (HR 1.7 [1.4–2.2]; p<0.001). Interestingly, the presence of brain metastases was more decisive for prognosis compared to the pattern of visceral distant metastasis according to AJCC in this model. CNS involvement independently increased the risk of death from melanoma by 1.6-fold [1.2–2.1];p = 0.001 in addition to the above mentioned serum markers, while the pattern of visceral involvement according to AJCC lost its significance and no longer represented an independent prognostic factor.

Finally, the treatment category was introduced in a third model. In the multivariate analysis no additional prognostic impact was observed according to the kind of treatment, while all other factors basically remained unchanged. In propensity modelling considering all confounding variables of model 3 (M category, LDH, presence of brain metastasis and type of treatment) a small adjustment of the hazard ratio for patients with elevated S100B serum levels was observed (adjusted HR 1.5 [1.2–1.9]; p = 0.002). Bootstrapping based on 1000 bootstrapping samples was performed to assess the generalizability of model 3 and showed a high level of internal validity (data not shown).

The independent negative impact of the presence of brain metastases, an elevated LDH and an elevated S100B observed in the second and third model was evident by stratifying patients according to the number of applicable unfavourable factors out of those three. We observed MSTs of 14 months in case of none, in contrast to 11, 6, and 4 months for patients with one, two, or all three factors applying (log rank p<0.001; Figure 1F).

## Discussion

In our analysis of 499 advanced melanoma patients with unresectable disease we found an independent prognostic impact of the S100B serum concentration in addition to the serum LDH. Furthermore, S100B was the best predictor for long-term survival. Five other studies compared both markers using multivariate analysis in patients receiving subsequent systemic treatment [10]–[14]. Deichmann *et al* also included a subset of patients who had subsequent surgery [14]. Both serum markers were significantly associated with survival in univariate analysis in all five studies. However, according to multivariate analyses, only either LDH or S100B remained a significant independent prognostic factor. S100B was superior compared to LDH in the studies of Egberts *et al* [10], [11], while the opposite was true in the other analyses [12]–[14]. Conflicting results might be caused by the low patient numbers between 61 and 145 in the above mentioned studies. The fact that in all studies only a single factor remained independently significant after multivariate analysis suggests a high degree of correlation between both serum markers.

In our analysis of 499 patients, which represent the thus far largest cohort of unresectable patients analysed for both serum markers, we surprisingly observed a substantial proportion of patients with discordant elevation of the serum markers. In 33.2% of patients with both markers available only S100B was elevated, and 3.7% had an isolated increase of LDH. The independent impact of both markers according to the multivariate analysis observed in our study can therefore be explained by the differential elevation of LDH and S100B in 36.9% of patients.

Many differences between both markers regarding tissue expression, biological function and others might also explain their differential increase observed in our patients. LDH is ubiquitously expressed in different tissues and elevated serum levels are mainly based on cell lysis. Elevation of serum LDH occurs in different types of cancer and is associated with high tumour burden, high cell turn-over of tumour cells or cell hypoxia-induced necrosis in fast growing metastasis. [7], [17], [18] An elevated serum level is an unspecific condition, which also occurs in many other - not cancer-related - inflammatory disorders, after physical tissue injury or after hypoxia-induced cell death (e.g. myocardial infarction). In contrast, S100B is relatively tissue specific and expressed in certain normal cells, most of which are originally derived from the neural crest, e.g. glial cells of the brain, melanocytes and chondrocytes. In addition, the corresponding cancer cells usually express S100B. S100B interacts with p53 and activates STK38/NDR1, being involved in cell survival and proliferation [19], [20] suggesting a functional capacity in cancer cells. It can be actively secreted [21] and is likewise elevated in patients without explicit cell turnover or damage like schizophrenia or depression [22]. These differences in the biology between S100B and LDH may explain the differential increase of serum levels in our patients and their independent prognostic impact.

Interestingly, the time point of analysis of prognostic factors in the course of disease seems to be important even after initial occurrence of distant metastases. In fact, the rate of elevated serum levels was increased in our patients receiving systemic treatments compared to patients at initial stage IV disease [3]. In this study, S100B serum levels were increased in 69% while this was only the case in 55% of patients after occurrence of the first distant metastasis. The relative increase was even more prominent for LDH with 40% in our study compared to 28% reported previously [3]. These variations during stage IV disease might explain, why S100B was shown to be superior compared to LDH to predict prognosis at earlier time-points, e.g. at the time of stage IV diagnosis or before metastasectomy of distant metastasis [3], [23], [24], while increasing impact is observed for LDH at later time-points e.g. at start of systemic treatment [12]–[14].

We additionally analysed the outcome according to the type of treatment. Even if differences in survival were detectable in univariate analysis, an independent effect was no longer observed applying multivariate testing. This is in agreement with a meta-analysis of 41 RTCs which showed no differences in survival comparing monochemotherapy, polychemotherapy, immunotherapy, and biochemotherapy [4]. This missing independent effect of different types of therapies if its relative impact is compared to other prognostic factors was not surprising because a high degree of selection bias has to be assumed. According to institutional and national guidelines patients receiving polychemotherapy were those with high tumor load or dynamic progression. In contrast, immunotherapies and biochemotherapies were mainly applied within clinical trials which often excluded patients with brain metastases or elevated LDH and therefore represent a cohort with a better a priori prognosis. Our finding is also in agreement with the fact, that until 2010 no systemic treatment showed an overall survival benefit in phase 3 RTCs. The situation changed after ipilimumab and vemurafenib became available. Both drugs improve overall survival, but nevertheless the clinical benefit is restricted to a small subset of patients for ipilimumab [25], [26] and it is questionable whether long-term survival can be induced by BRAF- or MEK inhibitors [27], [28]. In our study, the S100B serum level was stronger associated with long-term survival compared to the LDH serum level and the presence of brain metastases, both of which were other independent prognostic factors. Our observations provide a rationale to analyze the prognostic impact of S100B serum levels in patients treated with BRAF- or MEK inhibitors or by immune-checkpoint blockade and to investigate its role to select patients for these new therapeutic options. The observation of normal serum levels for both markers in patients who are in need for systemic therapy could be of clinical value especially in those with BRAF V600-mutant melanoma. In these patients, it might be beneficial to postpone treatment with vemurafenib or MEK-inhibitors and initially start with immunotherapy. Immunotherapy is characterized by longer treatment durations to achieve clinical responses as compared to inhibitor treatments. But also in BRAF V600 wild-type melanoma immunotherapy might be preferred compared to (poly-)chemotherapy as a first line treatment in patients with both markers within the normal range.

In conclusion, the S100B serum level predicts overall survival in unresectable melanoma patients receiving systemic treatment. Its relative impact is similar to LDH serum levels and the presence of brain metastases, both of which represented additional independent prognostic factors. Among those, S100B is most appropriate to predict long-term survival. The type of treatment applied between the years 2000 and 2010 did not influence prognosis. The role of S100B to select patients for treatment and to predict prognosis should be investigated in future studies in patients treated with new and more potent drugs in metastatic melanoma.
